# Supplementary material for: Topological gelation of reconnecting polymers
Source: Proc Natl Acad Sci U S A. 2022 Oct 24;119(44):e2207728119. doi: 10.1073/pnas.2207728119 (PMC9636914; doi:10.1073/pnas.2207728119)
Supplement: Supplementary File [file pnas.2207728119.sapp.pdf]

Topological Gelation of Reconnecting Polymers

Andrea Bonato,[1] Davide Marenduzzo,[1] Davide Michieletto,[1,2] and Enzo Orlandini[3]
[1]School of Physics and Astronomy, University of Edinburgh, Peter Guthrie Tait Road, Edinburgh, EH9 3FD, UK
[2]MRC Human Genetics Unit, Institute of Genetics and Cancer, University of Edinburgh, Edinburgh EH4 2XU, UK
[3]Department of Physics and Astronomy, University of Padova, Via Marzolo 8, I-35131 Padova, Italy and INFN, Sezione di Padova, Via Marzolo 8, I-35131 Padova, Italy

SIMULATION DETAILS

We performed constant-temperature (NVT) Molecular Dynamics simulations of confined bead-spring polymers made of beads of size σ and mass m in implicit heat bath (Langevin thermostat) at temperature T. The motion of the beads is described by the following set of Langevin equations,

$$m\frac{d^2\boldsymbol{r}_i}{dt^2} = -\zeta\frac{d\boldsymbol{r}_i}{dt} - \nabla U_i + \sqrt{2k_BT\zeta}\boldsymbol{f}_i, \quad \text{(S1)}$$

where $\boldsymbol{r}_i$ is the position of the center of mass of bead i, ζ is the friction and $\boldsymbol{f}_i$ is Gaussian white noise. U_i is the potential experienced by bead i, and is the sum of different contributions accounting for interactions and confinement. The equations of motion were evolved using a velocity-Verlet algorithm implemented by the LAMMPS package [1]; the integration time step was set to $0.001\tau_B$, where $\tau_B = \zeta\sigma^2/k_bT$ is the Brownian time of a bead of size σ. Excluded volume interactions between two unbonded beads are included via the Weeks-Chandler-Andersen (WCA) potential:

$$U_{WCA}(r) = \begin{cases} 4\epsilon\left[\left(\frac{\sigma}{r}\right)^{12} - \left(\frac{\sigma}{r}\right)^{6}\right] + \epsilon & r \leq r_c \\ 0 & r > r_c \end{cases}, \quad \text{(S2)}$$

where $r_c = 2^{1/6}\sigma$ and r is the distance between the centers of the beads. Consecutive beads are connected by finite-extension-nonlinear-elastic (FENE) bonds, introduced in the equations with the potential

$$U_{\text{FENE}}(r) = U_{WCA}(r) + \\ + \begin{cases} -0.5kR_0^2\ln\left(1-(r/R_0)^2\right) & r \leq R_0 \\ \infty & r > R_0 \end{cases}, \quad \text{(S3)}$$

where r is the separation between the the bonded beads, $k = 30\epsilon/\sigma^2$ is the spring constant and $R_0 = 1.5\sigma$ is the maximum extension of the bond. $\epsilon = k_BT$ is the energy unit.

The effect of stiffness is introduced by imposing a bending penalty acting on triplets of consecutive beads along the ring; this is achieved by introducing the Kratky-Porod potential

$$U_{KP}(\theta) = \frac{k_BTl_p}{\sigma}[1-\cos(\theta)], \quad \text{(S4)}$$

where l_p is the persistence length of the polymer and θ is the angle between the two bonds linking a triplet of neighbouring beads. The stiffness $K \equiv k_BTl_p/\sigma$ is a key parameter in our model.

The reconnecting rings are confined inside a sphere of radius R through a radial force field

$$F(r) = \begin{cases} -30\frac{\epsilon}{\sigma^3}(r-R)^2 & r \geq R \\ 0 & r < R \end{cases}, \quad \text{(S5)}$$

where r is the distance from the centre of the sphere.

In order to simulate elution experiments, we permeabilise the confinement sphere by using a rigid structure made of beads of size σ, whose interaction with the polymer beads is described by the WCA potential, (S2). The permeabilised sphere, which resembles a spherical grate, is built by overlapping rigid rings lying on planes perpendicular to one of the three axes of an orthogonal set.

To carry out reconnections with the LAMMPS software, we slightly modified the bond/swap fix, which implements the double bridging algorithm [2–4], to forbid the formation of linear chains made of 2 atoms, which can be formed by a self reconnection of a short loop of 4 consecutive atoms. More explicitly, the original algorithm allows the move $(\dots, i-1, i, i+1, i+2, \dots) \rightarrow (\dots, i-1, i+2, \dots) \cup (i, i+1)$ which we forbid to preserve the circular topology of all the polymers in the system. As described in the Materials and Methods section in the main text, an attempted reconnection move which would change the configuration of the system from ω to ω' (see Fig. 1A and section Model of the main text) is accepted with probability

$$p_{swap}(\omega', \omega) = \begin{cases} \exp(-\Delta E/k_BT) & \Delta E \geq 0 \\ 1 & \Delta E < 0 \end{cases}, \quad \text{(S6)}$$

where $\Delta E = E(\omega) - E(\omega')$ is the energy difference between ω and ω'.

DETAILED DERIVATION OF THE FREE ENERGY OF RECONNECTING RINGS AT EQUILIBRIUM

In this section we derive the free energy of the reconnecting rings reported in the main text. For simplicity,

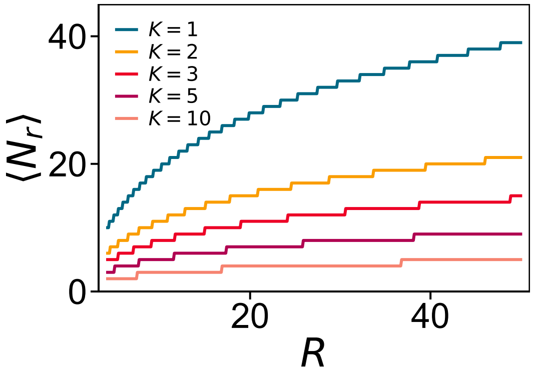

Figure S1. Average number of rings as a function of the confinement radius, predicted from Eq. (S13) with $\lambda = 5.3$ and $K = 1, 2, 3, 5$ and 10.

let us suppose that in the long time limit there are m rings of equal contour length $l_i = l = N\sigma/m = L/m$ where N is the total number of monomers in the system and σ is the step length (i.e. the distance between two consecutive monomers). If l_p is the persistence length, the corresponding bending energy is

$$U = k_B T \sum_{i=1}^{m} \frac{2 l_p \lambda \pi^2}{l_i} \simeq m k_B T \frac{2 l_p \lambda \pi^2 m}{L} \,, \tag{S7}$$

while an estimate of the entropy is given by $S = k_B \ln Z$, where Z is the number of configurations of the system. We point out that, in Eq. (S7), we assumed the bending energy of each ring of the system to be $k_B T \frac{2 l_p \lambda \pi m^2}{l_i}$. This is an approximation of the bending energy of ring polymers of any contour length l_i, which was set such that its functional form coincides with the bending energy of short rings (with length comparable to l_p). λ is a fitting parameter To estimate Z we suppose that the rings are independent. This gives

$$Z = \sum_{l_1,\dots,l_m} f(l_1, \dots, l_m) \prod_{i=1}^{m} Z^1(l_i), \tag{S8}$$

where the sum is over all the possible values of the m lengths l_i (which are multiples of σ compatible with $\sum_{i=1}^{m} l_i = L = N\sigma$ and $l_i \geq 3\sigma \; \forall i$), f accounts for all the possible ways N monomers are distributed between the m rings (i.e. reconnection), and $Z^1(l_i)$ is the number of configurations for a ring with length l_i. This number is not known exactly but if we restrict ourselves to the case of freely-jointed linear chains with Kuhn length $2l_p$ and confined in a space of volume $V = R^3$ we have [5]

$$Z^1(l_i) = \exp\left(-\frac{l_p \pi^2}{V^{\frac{2}{3}}} l_i + \ln(V) + \ln(2^9/\pi^6) \right). \tag{S9}$$

From (S9) we then have

$$\prod_{i=1}^{m} Z^1(l_i) = \exp\left(-\frac{l_p \pi^2}{V^{\frac{2}{3}}} L + m\ln(V) + m\ln(2^9/\pi^6) \right). \tag{S10}$$

On the other hand,

$$\sum_{l_i,\dots,l_m} f(l_i, \dots, l_m) \approx \{N, m\} \left[\frac{1}{2} \left(\frac{N}{m} \right)! \right]^m , \tag{S11}$$

where $\{N, m\}$, is the Stirling number of the second kind and counts all the possible ways to partition N monomers into m groups while $\left[\frac{1}{2}\left(\frac{N}{m}\right)!\right]$ is the number of permutations of the monomers of a linear chain of length $l = \frac{N\sigma}{m}$. This gives

$$\frac{S}{k_B} = \ln(\{N, m\}) + m\ln\left[\left(\frac{N}{m}\right)!\right] - \frac{l_p \pi^2}{V^{\frac{2}{3}}} L + m\ln\left(\frac{V 2^8}{\pi^6}\right). \tag{S12}$$

The free energy of the system $F = U - TS$ is then given by

$$\frac{F}{k_B T} = \frac{2 l_p \lambda \pi^2 m^2}{L} + m\ln(m) - m - m\ln\left(\frac{V 2^8}{\pi^6}\right) + const\,. \tag{S13}$$

In Fig. S1 we plot the average number of rings $\langle N_r \rangle$ as a function of the confinement radius R, predicted from Eq. S13 with $\lambda = 5.3$. The figure shows that gelation can be achieved by decreasing R.

ADDITIONAL RESULTS

Robustness against initial conditions

Figure Fig. S2 shows that starting the system from different conditions (m chains with n beads with fixed total number of beads $N = n \times m = 10000$) yields the same large time steady state.

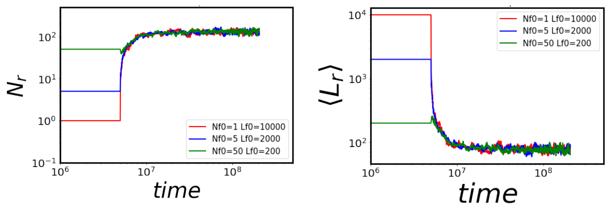

Figure S2. Behaviour of the number and length of the rings over time for systems with a total number of beads $N = 10000$, but starting from different conditions (see legend).

The transition from unlinked to linked is present for flexible rings at large enough volume fraction

In Figure S3 we show that the crossover between a fluid of unlinked rings and a gel of linked rings occurs

also for flexible rings at large enough densities. In the main text we report that the transition is around $K = 2 - 3$ for fixed volume fraction $\phi \simeq 0.37$ (monomer number density $\rho = 0.7\sigma^{-3}$). In Figure S3 we show that at fixed $K = 1$ we still observe a crossover between the two regimes with evidence of a percolating network of linked rings at $\phi \simeq 0.4 - 0.5$. Accordingly, we argue that nematic ordering does not play a major role in driving the topological transition.

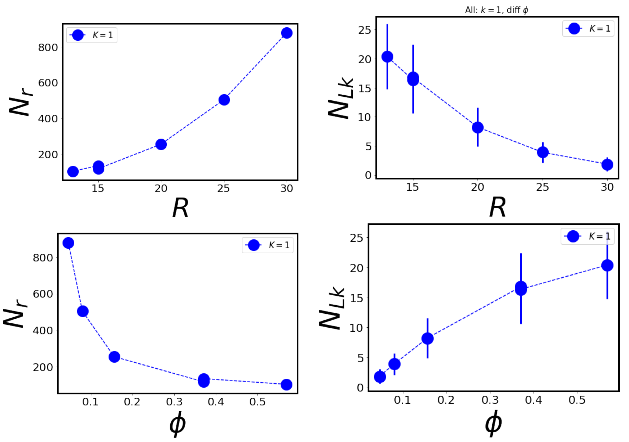

Figure S3. Behaviour of the average number of rings N_r and number of linked pairs N_{lk} at equilibrium for fixed $K = 1$ and varying confinement. Top row shows the behaviour as a function of confinement radius R, the bottom row shows the same values but as a function of the volume fraction ϕ. The figure shows results for systems with a total number of beads $N = 10000$.

Geometry of reconnecting rings: length and spatial distributions

Here we complement the results on the geometrical properties of the reconnecting polymers reported in the main text. We look first at the length, or size, distribution of the rings at the steady state for different bending rigidity, see Fig. S4. As the stiffness of the rings grows, the probability of observing large rings increases. Additionally, these distributions exhibit a power law decay for large lengths with exponent close to -1 for all values of the stiffness parameter K (Fig. S4F). Simulations with a larger N by an order of magnitude yield similarly broad ring length distributions (Fig. S5).

To study whether rings segregate, in Fig. 2G of the main text, we plot the degree of mixing of a ring of length s as a function of s. It is the ensamble average of the observable $d^m(s)$, which, for a configuration of N_r rings with lengths l_i, $i = 1, \ldots, N_r$, is defined as

$$d^m(s) = \frac{\sum_{i=1}^{N_r} \delta_{l_i s} \sum_{j=1}^{l_i} \chi(n_{R_{sp}}(i,j))}{\sum_{i=1}^{N_r} l_i \delta_{l_i s}}, \quad \text{(S14)}$$

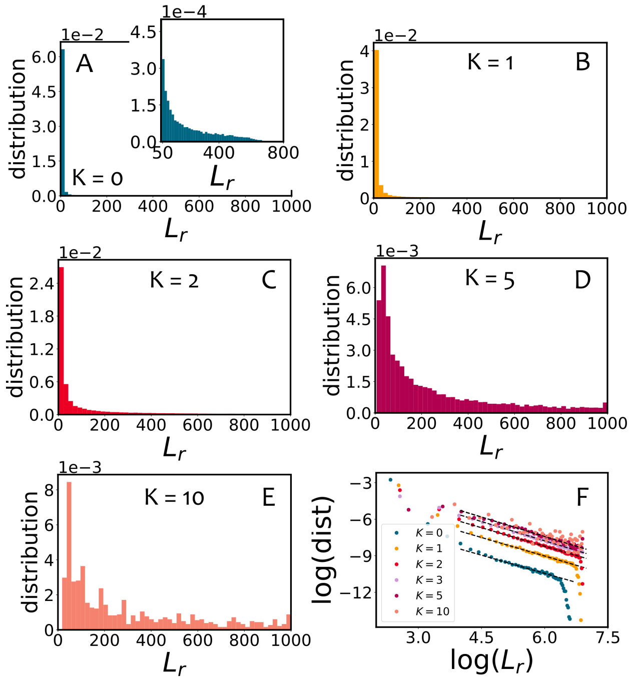

Figure S4. **(A-E)** Length distribution of reconnecting rings for $K = 0$, $K = 1$, $K = 2$, $K = 5$ and $K = 10$, and $N = 1000$. **(F)** Log-log plot of the length distribution for $K = 0$, $K = 1$, $K = 2$, $K = 3$, $K = 5$ and $K = 10$. The fitting curves are lines with slope -1.

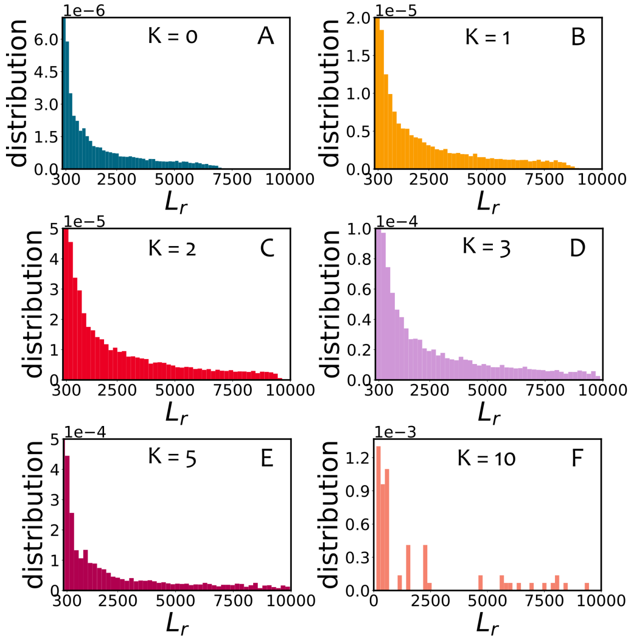

Figure S5. **(A-E)** Length distribution of large reconnecting rings for $K = 0$, $K = 1$, $K = 2$, $K = 3$, $K = 5$, and $N = 10000$. Zoom in on the region $L_r \geq 250$. **(F)** Length distribution for $K = 10$ and $N = 10000$.

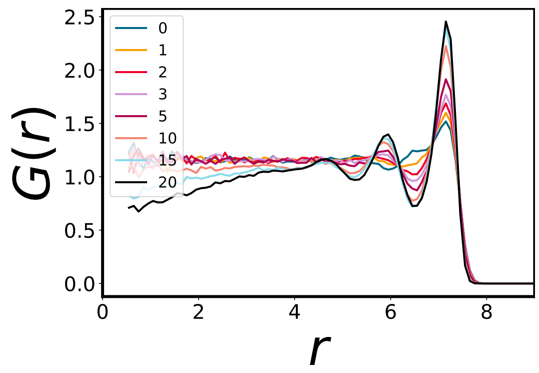

Figure S6. Normalised radial distribution of all monomers for $K = 0$, $K = 1$, $K = 2$, $K = 3$, $K = 5$, $K = 10$, $K = 15$ and $K = 20$.

where

$$n_{R_{sp}}(i,j) = \sum_{\substack{m=1\\ m\neq i}}^{N_r} \sum_{n=1}^{l_m} \chi\left(R_{sp} - |\mathbf{r}_{ij} - \mathbf{r}_{mn}|\right) \qquad \text{(S15)}$$

is the number of beads belonging to rings $m = 1, \ldots, N_l$, $m \neq i$, inside a sphere of radius R_{sp} centered in $\mathbf{r}_{ij}$, the position of the $j-$th monomer of the $i-$th ring. $R_{sp} = 1.8\sigma$. In Eqs. S14 and S15, δ is the Kronecker delta,

$$\delta_{ij} = \begin{cases} 1 & i = j \\ 0 & i \neq j \end{cases} . \qquad \text{(S16)}$$

and χ is defined as

$$\chi(x) = \begin{cases} 1 & x > 0 \\ 0 & x \leq 0 \end{cases} . \qquad \text{(S17)}$$

Another quantity of interest is the normalised radial density of all monomers, $G(r)$. This is reported in Fig. S6 for different values of K. We note that the distributions are non-uniform and characterised by a peak close to the surface of the confinement sphere, which becomes more pronounced as K increases. This distribution is reminiscent of the density profile observed at an interface between a confining wall and a colloidal fluid.

Finally, in Fig. S7(A-C) we report a typical snapshot of steady state configurations of reconnecting rings simulated at stiffness $K = 1, 3, 5$ respectively. Note that as rings becomes more flexible they shorten and are depleted from the interior of the confining sphere. This is also confirmed by the corresponding radial distribution map $G(r)$ reported in Fig. S7 (D-F).

Detecting topologically linked structures

As mentioned in the Materials and Methods section of the main text, the pairwise topological complexity of the system of rings is estimated by computing the Gaussian linking number for each pair of rings γ_i and γ_j.This is given by

$$LK(\gamma_i,\gamma_j) = \frac{1}{4\pi}\oint_{\gamma_i}\oint_{\gamma_j} \frac{\boldsymbol{r}_i - \boldsymbol{r}_j}{|\boldsymbol{r}_i - \boldsymbol{r}_j|^3} \cdot (d\boldsymbol{r}_i \times d\boldsymbol{r}_j) . \qquad \text{(S18)}$$

For each pair (i, j) and sampling time t we defined

$$\chi(i,j)(t) = \begin{cases} 1 & |LK(\gamma_i,\gamma_j)|(t) > 0.5 \\ 0 & |LK(\gamma_i,\gamma_j)|(t) \leq 0.5 \end{cases} , \qquad \text{(S19)}$$

and, by summing over all pairs, we obtained the number of linked pairs of a given configuration at time t:

$$N_{LK}(t) = \frac{1}{2}\sum_{i,j}^{N_r(t)} \chi(i,j)(t) , \qquad \text{(S20)}$$

where $N_r(t)$ is the number of rings at time t. The function $N_{LK}(t)$, averaged over several trajectories, is reported in Fig. 4A of the main text. Similarly, we computed the total absolute value of the linking number of a configuration as

$$|LK|(t) = \frac{1}{2}\sum_{i,j} |LK(\gamma_i,\gamma_j)|(t) \qquad \text{(S21)}$$

and its average over several trajectories, is reported in Fig. 4B of the main text. In the long time limit the above quantities reach a plateau indicating that the systems have reached a steady state regime. By performing a further average over configurations in this regime we obtained the steady state estimates $\langle N_{LK}\rangle$ and $\langle |LK|\rangle$ that are reported, as a function of the persistence length K, in Fig. 4C and 4D, respectively.

In Fig. S8 we report the distribution of the absolute value of the linking number between pair of rings (γ_1, γ_2) for different values of the stiffness. This pairwise measure of link complexity shows that fully flexible rings are mostly unlinked, while, as K increases, the link spectrum broadens suggesting a steadily increase of the link complexity for more rigid confined reconnecting rings. Notably, for $K = 15$ there are configurations in which a pair of reconnecting rings has with $|LK| = 10$.

Size of clusters of linked rings

To characterise the topological complexity of the reconnecting rings in steady state beyond the pairwise linking description we look for the presence and statistics of networks of pairwise topologically linked rings. In this description the nodes of the network $i = 1, \cdots N_r$ represent the rings of the system while the presence of link connecting the pair of nodes (i, j) indicates that the corresponding pair of rings (i,j) have non trivial linking number. Within this picture unlinked rings correspond to isolated nodes while a cluster of m connected nodes corresponds

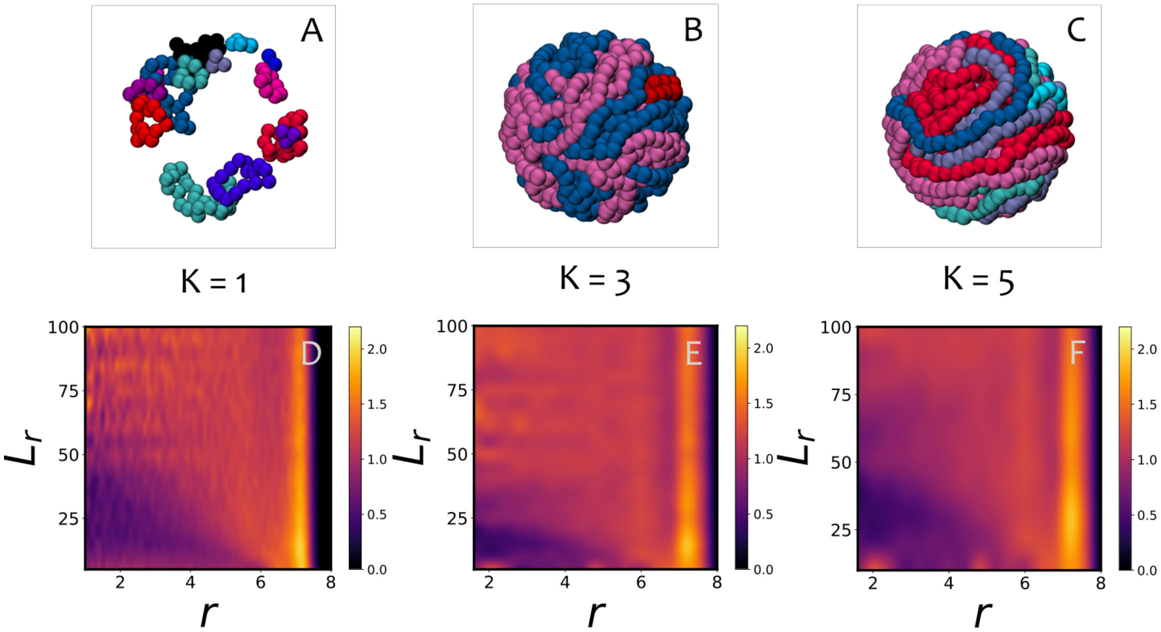

Figure S7. **(A-C)** Snapshot from simulations with $K = 1$, $K = 3$ and $K = 5$. Different colours are used to identify each individual ring. For $K = 1$, only short rings ($L_r < 30$) are shown. **(C-E)** Radial distribution of monomers in rings of length L_r as a function of r, the distance from the centre of the confinement sphere, and L_r for $K = 1$, $K = 3$ and $K = 5$.

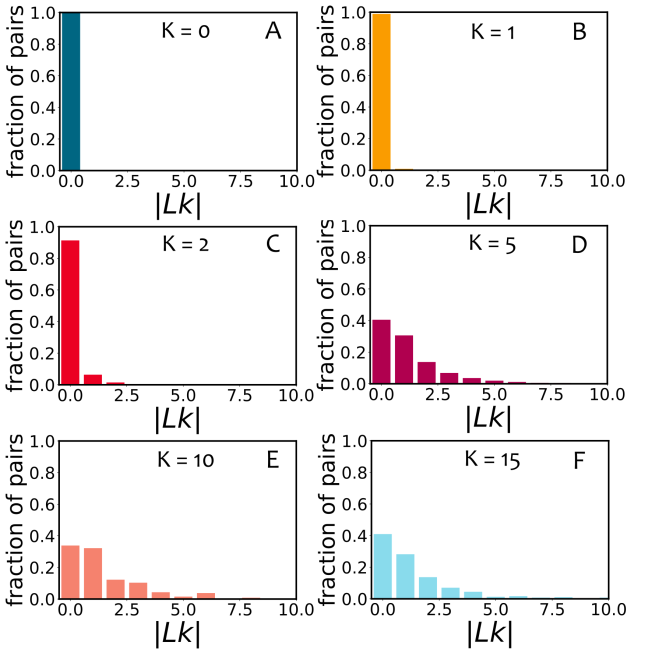

Figure S8. **(A-F)** Distribution of the absolute value of the linking number of pairs of reconnecting rings for $K = 0$, $K = 1$, $K = 2$, $K = 2$, $K = 5$, $K = 10$ and $K = 15$.

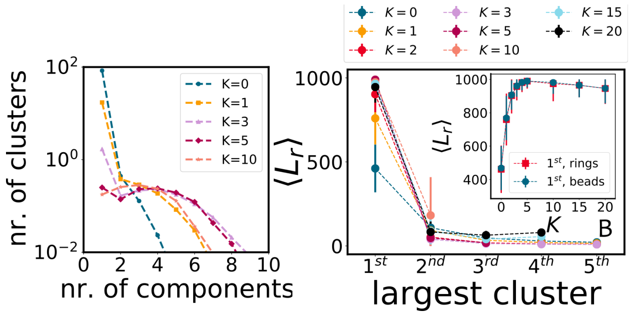

Figure S9. **(A)** Fraction of observed clusters of linked rings as a function of the number of components for $K = 0$, $K = 1$, $K = 3$, $K = 5$ and $K = 10$. **(B)** Average number of monomers involved in the five largest (i.e. with the most monomers) clusters of a sampled configuration. The inset displays the number of monomers involved in the cluster with the most components (red) and in the cluster with the most monomers (blue) as a function of K.

in real space to m rings that are interlocked by topological links. Examples of topological clusters and the corresponding configurations are reported in Fig. 5B of the main text.

In Fig. S9A we report the observed number of clusters with a given number of components, that is the number of rings that are topologically connected by linking. It can be seen that softer rings (smaller values of K) are mostly unlinked while for more rigid ones clusters of multicomponent links are more frequent. Noticeably, largest clusters are observed for intermediate values of K, a result that agrees with the non monotonic behaviour observed in Fig. 6 of the main text.

In Fig. S9B we report the mean number of monomers involved in the largest clusters (i.e. with the largest number of rings) for different values of the stiffness param-

eter K. Notably, even for fully flexible rings ($K = 0$), when the mean number of components of the largest cluster is about 2 (two-components links), the number of monomers involved in the largest cluster is about half of the total. In other words for the system of flexible rings the few observed clusters are mostly two-component links whose rings have a non negligible contour length. The inset plot compares the number of monomers of the cluster with the largest number of components with the number of monomers of the cluster with the largest number of monomers. Since the two curves are almost identical for all values of K, we argue that the cluster with the most component is also the cluster with the most monomers.

Detection of knotted structures

Sufficiently long products can be knotted and due to confinement these knots can be also quite complex. In order to detect the knotted state of a ring we first simplify it by using local deformations [6]. Of the simplified ring configurations we chose the projection with the minimum number of crossings produced by the smoothing scheme and encoded it in terms of the Dowker code and then fed this code to the Knotscape [7] program to detect the knot type. Some of the knots detected are those reported in Fig. S10.

Escape dynamics from a permeabilised sphere, with reconnection

In the main text, as an indirect measure of the topological gel state of the reconnecting rings, we looked at the escape dynamics of the products of reconnection after permeabilisation of the confining sphere. More precisely, once a system of reconnecting rings had reached a steady state the reconnection process was switched off, many holes drilled in the confining sphere (permeabilisation) and the number of monomers still inside the sphere was monitored as a function of time.

A different scenario is observed if the reconnection process is still active during elution. In Fig. S11A we report the number of monomers still inside the permeabilised sphere as a function of time for fully flexible rings ($K = 0$). Strikingly, the number of monomers inside the sphere decreases until it reaches ~ 14, which is the value expected in the case of monomers uniformly distributed inside the simulation box. The decay is well fitted through the whole range of time by a stretched exponential. This is mostly due to the fast escape dynamics of the short rings produced by reconnection: as the sphere depletes, the reconnecting polymers left inside progressively disassemble into little fragments. Short rings can then easily escape confinement, and distribute themselves uniformly in the simulation box.

Different is the outcome for a system of stiffer rings ($K = 5$), as shown in Fig. S11B. In this case, even with the reconnection process still active, very few rings are small enough to rapidly pass through the pores of the permealised sphere and the elution dynamics is mostly governed by the translocation of long and possibly linked rings through a pore. This is in general a very slow process, but in this case we note that the pores may act in synergy with reconnection by forcing two segments of the same ring to be proximate in space, increasing the probability of occurrence of reconnection events that eventually simplify the long rings while they exit the sphere.

We note that the two $n(t)$ curves behave qualitatively similarly to the correlation function describing the dynamics of neighbour exchange in liquids and glasses [8], thereby reinforcing our characterisation of the transition which we observe as gelation.

[1] A. P. Thompson, H. M. Aktulga, R. Berger, D. S. Bolintineanu, W. M. Brown, P. S. Crozier, P. J. in 't Veld, A. Kohlmeyer, S. G. Moore, T. D. Nguyen, R. Shan, M. J. Stevens, J. Tranchida, C. Trott, and S. J. Plimpton, Comp. Phys. Comm. **271**, 108171 (2022).

[2] N. C. Karayiannis, A. E. Giannousaki, V. G. Mavrantzas, and D. N. Theodorou, Journal of Chemical Physics **117**, 5465 (2002).

[3] N. C. Karayiannis, V. G. Mavrantzas, and D. N. Theodorou, Physical Review Letters **88**, 4 (2002).

[4] R. Auhl, R. Everaers, G. S. Grest, K. Kremer, and S. J. Plimpton, Journal of Chemical Physics **119**, 12718 (2003), arXiv:0306026 [cond-mat].

[5] S. Edwards and K. Freed, Journal of physics A: General physics **2**, 145 (1969).

[6] C. Micheletti, D. Marenduzzo, E. Orlandini, and D. Summers, J. Chem. Phys. **124**, 064903 (2006).

[7] J. Hoste and M. Thistlethwaite, "Knotscape," `http://www.math.utk.edu/~morwen/knotscape.html`.

[8] D. Michieletto, E. Orlandini, and D. Marenduzzo, Physical Review X **6**, 041047 (2016).

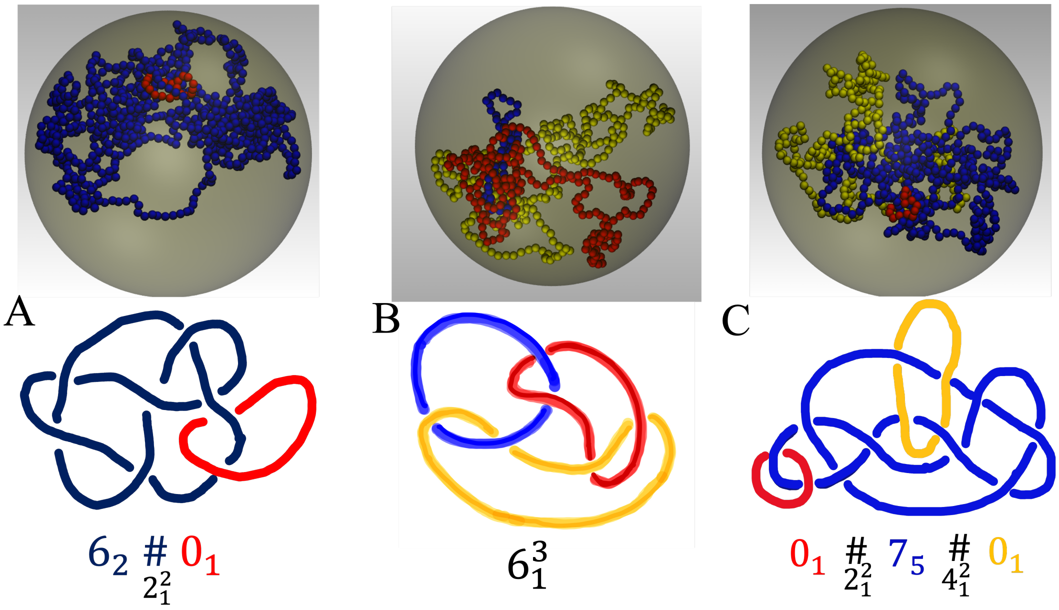

Figure S10. **(A-C)** Examples of configurations that are topological linked and knotted for a system of reconnecting rings with $K = 2$, $R = 15\sigma$ and $N = 10000$. **(A)** A Hopf-linked pair of rings in which one component is knotted with knot type 6_1. The size L_r of the two rings are $n_1 = 72$ and 796 respectively for the unknot and the 6_1 knot. **(B)** A 3-components prime link known as the 6^3_1 link. Each component is unknotted. The length of the rings are $L_r^{(1)} = 58$ (blue), $L_r^{(}2) = 243$ (red) and $L_r^{(}3) = 257$ (yellow). (C) A very complex instance of 3-components link in which the longest ring (blue ring, $L_r = 6445$) is a 7_5 knot that is Hopf-linked to an unknot (red ring, $L_r = 21$) and forms a Solomon link with the third unknotted component of length $L_r = 1122$ (yellow ring).

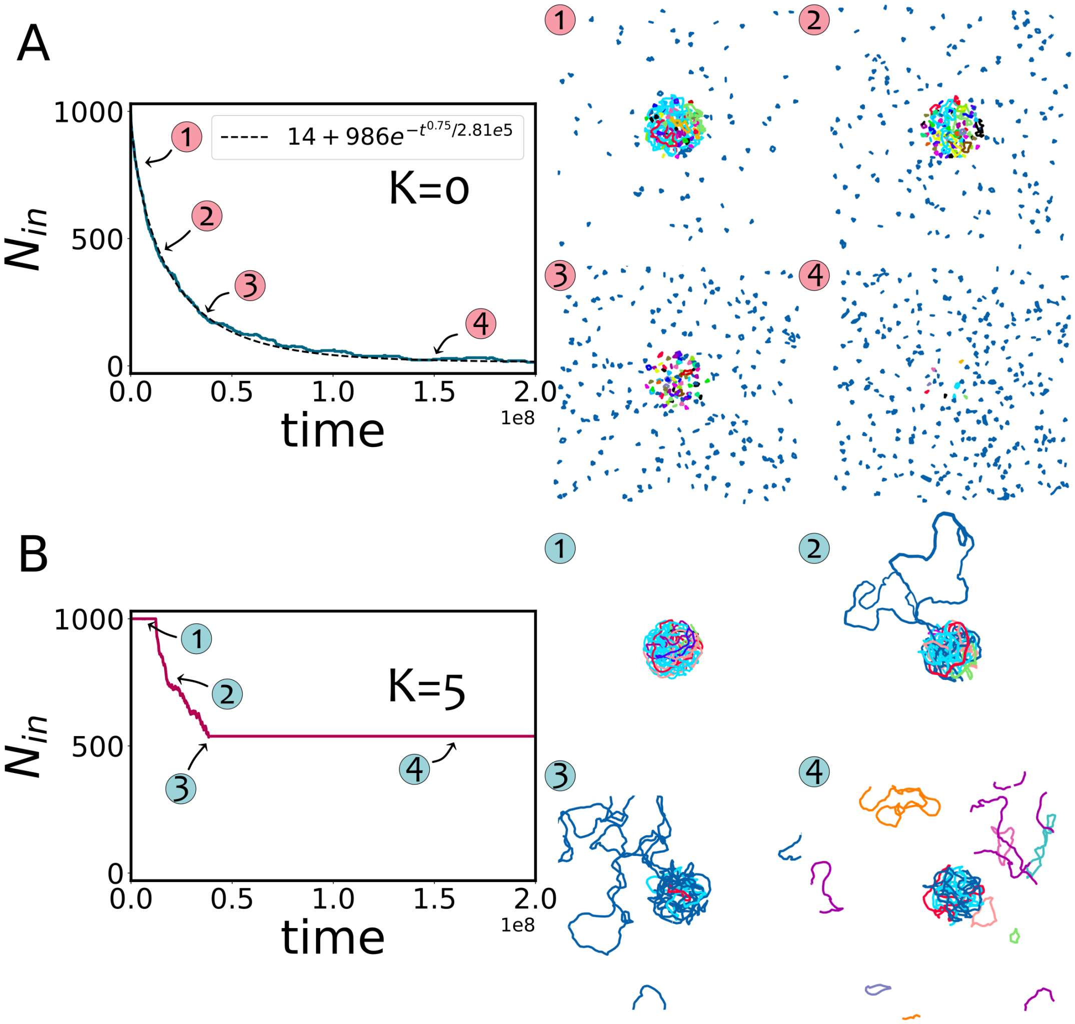

Figure S11. Simulations of reconnectiong rings escaping from a permeabilised sphere of radius $R = 9$. Unlike the cases presented in the main text reconnection events can occur is active throughout the elution process. **(A)** Number of monomers still inside the sphere as a function of time for a system of fully flexible rings ($K = 0$), and snapshots taken at different times. Rings which escaped confinement are coloured in cyan, different confined rings are drawn with different colours. **(B)** Number of confined monomers as a function of time for $K = 5$ and corresponding snapshots. Note that these simulations are performed in a periodic box, hence why some rings appear to have free ends.
